# Wearable Inertial Sensor System towards Daily Human Kinematic Gait Analysis: Benchmarking Analysis to MVN BIOMECH

**DOI:** 10.3390/s20082185

**Published:** 2020-04-12

**Authors:** Joana Figueiredo, Simão P. Carvalho, João Paulo Vilas-Boas, Luís M. Gonçalves, Juan C. Moreno, Cristina P. Santos

**Affiliations:** 1 Center for MicroElectroMechanical Systems (CMEMS), Industrial Electronics Department, University of Minho, 4800-058 Guimarães, Portugal; simaopedrocarvalho17@gmail.com (S.P.C.); lgoncalves@dei.uminho.pt (L.M.G.); cristina@dei.uminho.pt (C.P.S.); 2 Faculty of Sport, CIFI2D, and Porto Biomechanics Laboratory (LABIOMEP), University of Porto, 4200-450 Porto, Portugal; jpvb@fade.up.pt; 3 Neural Rehabilitation Group, Cajal Institute, Spanish National Research Council, 28002 Madrid, Spain; jc.moreno@csic.es

**Keywords:** inertial sensors, gait analysis, human daily motion analysis, sensor fusion, wearable sensors

## Abstract

This paper presents a cost- and time-effective wearable inertial sensor system, the InertialLAB. It includes gyroscopes and accelerometers for the real-time monitoring of 3D-angular velocity and 3D-acceleration of up to six lower limbs and trunk segment and sagittal joint angle up to six joints. InertialLAB followed an open architecture with a low computational load to be executed by wearable processing units up to 200 Hz for fostering kinematic gait data to third-party systems, advancing similar commercial systems. For joint angle estimation, we developed a trigonometric method based on the segments’ orientation previously computed by fusion-based methods. The validation covered healthy gait patterns in varying speed and terrain (flat, ramp, and stairs) and including turns, extending the experiments approached in the literature. The benchmarking analysis to MVN BIOMECH reported that InertialLAB provides more reliable measures in stairs than in flat terrain and ramp. The joint angle time-series of InertialLAB showed good waveform similarity (>0.898) with MVN BIOMECH, resulting in high reliability and excellent validity. User-independent neural network regression models successfully minimized the drift errors observed in InertialLAB’s joint angles (NRMSE < 0.092). Further, users ranked InertialLAB as good in terms of usability. InertialLAB shows promise for daily kinematic gait analysis and real-time kinematic feedback for wearable third-party systems.

## 1. Introduction

Human gait analysis, namely the kinematic data, has manifold applications, as follows. First, it has the potential to be applied as an automatic assessment tool for motor disorders to foster better treatment decisions. Second, to design personalized gait therapies. Third, to recognize walking risk situations, and to support the clinical motor diagnosis [1,2]. 

Current challenges include the development of wearable motion labs with unobtrusive, low-cost, and effective wearable sensor systems for all-day and any-place gait monitoring without interfering with the user’s movement [1,3]. Research contributions related to the ambulatory human kinematic gait analysis may involve inertial sensor-based systems with inertial measurement units (IMUs) [1]. Low-cost IMUs-based sensor system showed excellent performance for joint angle gait analysis with lower errors than the low-cost vision-based gait capture systems [4]. 

There is growing interest in developing low-cost, wearable IMU-based systems for kinematic gait analysis, including orientation estimation [5,6,7,8] and gait event analysis [5], and their integrating into third-party systems [6,9]. Nonetheless, the systems proposed in the research community have to deal with challenges, as follows: (i) automatic, user-independent calibration to avoid the use of time-consuming calibration methods [5,8], (ii) time-effective computational tools, eventually combined with biomechanical models, for the real-time orientation estimation [10], (iii) technical matters to deal with sensor’s misalignments [11,12], and (iv) to assess the reliability of the inertial sensor system for gait analysis in daily and non-structured conditions for inferring its real-world application validity. 

Related studies that developed wearable IMU-based systems for kinematic gait analysis, including segment orientation and/or joint angle estimation [5,6,11,13], face a limited validation analysis concerning daily walking conditions. The study [6] presents a 7 IMU-based system, either for integration into lower limb exoskeletons or human gait kinematic analysis, using Kalman filtering and the factored quaternion algorithm for orientation estimation. The validation of the wearable inertial measurement system included flexion/extension isolated motions of lower limb joints and 30 walking steps. The performance analysis of the wearable IMU-based system proposed in [5] was limited to four strides. This system integrates six IMUs (three-dimensional (3D)-gyroscopes and two-dimensional-accelerometers) placed on the shank, thigh, and foot segments, aiming lower limb orientation estimation through mathematical integration of the angular velocity measured. Kardos et al. [13] proposed a seven IMU sensor array system designed for kinematic gait sensing, namely joint angle estimation based on fusion methods (complementary and Kalman’s filters). However, this study did not disclose results concerning the system’s effectiveness for gait analysis. Tadano et al. [11] presented a wearable sensor system consisting of seven sensors (3D accelerometer and gyroscope) for lower limb orientation estimation using quaternion. Experiments were conducted indoors on a straight flat floor with five healthy volunteers. Additionally, Kyrarini et al. [4] asked the participants to walk in a straight line for 2.5 m to compare the performance of vision-based and commercial IMU-based systems for joint angle gait analysis.

On the other hand, commercial IMU-based solutions such as MVN BIOMECH (Xsens, Netherlands), RIABLO (CoReHab, Italy), G-walk (BTS Bioengineering Corp., Quincy, Massachusetts, USA) (i) are high-cost systems, (ii) usually require non-wearable processing units to run the software interfaces, (iii) do not offer a fully wearable integration into a further sensor and actuation systems, and (iv) do not directly and efficiently provide the real-time kinematic data to third-party devices or algorithms (for instance, techniques for human motion intention recognition and motor diagnosis).

This study holds three main goals. First, we developed a wearable inertial sensor system, the InertialLAB, for the real-time tracking of 3D-angular velocity and 3D-acceleration up to 6 lower limbs and trunk segment, and joint angle measures in the sagittal plane up to 6 joints. The kinematic data monitored by InertialLAB aimed at a holistic gait analysis application every day and anywhere, such as the gait event analysis reported in [14] and human locomotion intention decoding [15]. It can be used for healthy and pathological gait assessment, either for preventive healthcare or for the evaluation of rehabilitation processes and assisted gait conditions by a wearable exoskeleton [16].

InertialLAB was designed to be cost-, time-effective, and easily calibrated. It includes an automatic and user-independent calibration with a minimum-to-null effort for the user and assessor. InertialLAB has an advantage over existing solutions by avoiding the commonly applied manual, time-consuming, and subject-specific calibration. Moreover, InertialLAB encompasses up to seven small, lightweight IMUs placed on the back, thigh, shank, and foot segments, as suggested by [9,17] for gait analysis applications. The developed software interfaces running up to 200 Hz for real-time (i) monitoring and providing kinematic data to third-party systems, (ii) segment orientation determination using inertial data fusion-based methods, the complementary filter and Kalman filter, and (iii) joint angle estimation using a trigonometry-based method dependent on the segments’ orientation and segment-joint relations in the calibration. InertialLAB attempts to pursuit the limitations found in similar commercial systems. It is a low-cost gait data capture system that includes a modular and open-architecture to enable both stand-alone use and direct integration into third-party systems, such as robotic assistive devices [16]. Consequently, compared to similar commercial systems, InertialLAB can timely provide real-time kinematic data to third-party wearable devices or algorithms. This main innovative feature of InertialLAB is determinant for yielding personalized therapies concerning the user’s actual kinematic state. Additionally, the fairly simple software interfaces of InertialLAB make it a low computational load system to enable their execution into wearable processing units, advancing commercial systems that require non-wearable processing units. Thus, InertialLAB offers a fully wearable solution for more practical daily use.

The second goal covers a benchmarking analysis of InertialLAB against the MVN BIOMECH [18], a well-established commercial wearable system for human kinematic gait analysis [1,12]. This benchmarking analysis involved heterogenous gait cycles from healthy subjects walking varying gait speed and terrain (flat, ramp, and stairs) and including turns. This validation innovates the actual literature works [4,5,6,11,13], as follows: (i) by extending the joint angle analyzes with the 3D-angular velocity and 3D-acceleration analyses and (ii) including heterogenous gait patterns from more subjects and more non-structured real-world scenarios (flat surfaces, stairs, and ramps). The validation enables us to better assess the reliability, repeatability, and practical evidence of InertialLAB, as a low-cost wearable inertial sensor system for daily kinematic gait analysis. When compared to related studies [4,5,6,11,13], this work extends the InertialLAB’s validation by performing a usability assessment using the system usability scale [19].

Lastly, we explored the hypothesis that a machine learning-based regression model would improve the InertialLAB’s accuracy for joint angle estimation with minimal drift bias. This approach potentially eliminates the need for high-complex biomechanical models for reliable joint angle estimation. For this purpose, we compared the accuracy of different machine learning-based regression models, namely neural network (NN), decision tree, and support vector machine (SVM), to identify a well-fitted model for improving the accuracy of InertialLAB for joint angle estimations.

## 2. Materials and Methods

### 2.1. InertialLAB Requirements

InertialLAB’s design addressed six main requirements. First, the hardware interfaces should embed low-cost and efficient electronic components to produce a cost-effective, low-power consumption system. Second, InertialLAB should be an easily wearable system to cover the 10th-to-90th percentile of the male/female population to increase the user’s usability. Third, the system should include a time-effective communication protocol aiming for the inclusion of up to seven low-power, light-weight, cheap IMUs to provide the necessary data for gait analysis [9]. Fourth, software routines should follow a modular, open-architecture to provide real-time information to third-party systems and allow a relatively high sampling frequency (≤200 Hz) to meet the computational requirements of high-performance tools, such as the motion intention recognition and control architectures [20]. Fifth, InertialLAB should endow a prompt calibration routine with a minimum-to-null effort. Lastly, the system’s autonomy should last for at least eight hours, accommodating prolonged recording sessions.

### 2.2. InertialLAB: Hardware-In-The-Loop

**Figure 1:**
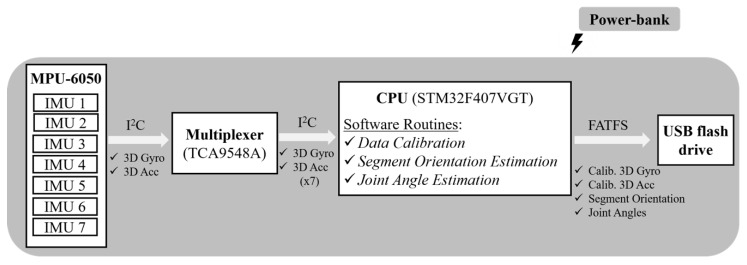
Block diagram of InertialLAB.

Figure 1 illustrates the block diagram of InertialLAB, highlighting its architecture and the used hardware modules. InertialLAB is scalable as desired up to seven IMUs and includes a wearable central processing unit (CPU). As proposed in [13], each IMU consists of the MPU-6050 (InvenSense, Boston, MA, USA) [21] that combines a 3-axis MEMS accelerometer (±8 g) and a 3-axis MEMS gyroscope (±2000°/s) for the kinematic data acquisition. We selected this IMU given its small size (15 × 20 × 2 mm) and weight (0.009 kg), low admissible current consumption (3.8 mA), and linear behavior in the operating weather conditions (−40 °C to +85 °C). MPU-6050 endows peripheral controller and I^2^C interface for fast communication mode to 400 kHz. Additionally, this sensing unit has been pointed out as the world’s first motion tracking device designed for the low power, low cost, and high-performance requirements of wearable sensors [21], as specified for InertialLAB. We did not include a magnetometer to avoid the complications related to the magnetic field since hard iron effects can be found and compromise the InertialLAB’s application in outdoor and rehabilitation scenarios as inner prostheses and treadmill [8].

We designed a multi-channel board (80 × 80 × 25 mm) to include the electronic components needed for multi-channel recording up to seven IMUs. The multi-channel board integrates the TCA9548A I^2^C multiplexer (it gets up to 8 same-address I^2^C devices hooked up to one microcontroller) to manage the information collected by seven IMUs until the CPU. MPU-6050 communicates with CPU through I^2^C protocol (up to 400 kHz, enough for the proposed application). For the I^2^C, we used USB cables to enable an easy plug and unplug solution for real-life gait sensing. In this development stage, we selected a wired communication protocol to guarantee the requirements of strict determinism, time-effective (up to 200 Hz), and optimized data transfer.

Furthermore, we incorporate the STM32F407VGT (STMicroelectronics, Geneva, Switzerland) with an ARM^®^ Cortex^®^-M4 32-bit core (running at 168 MHz) to fulfill real-time and portability requirements. This CPU (80 × 100 × 25 mm) has the resources required for a time-effective (up to 200 Hz) acquisition, processing, and transmission of the data, and to execute the set software interfaces. Additionally, it communicates with an attached USB flash drive (4 GB of storage capacity, write speed of 8 MB/s) using the FATFS library to store the collected data (3D-gyroscope and 3D-accelerometer data, and the segment and joint angles) for more than eight hours for offline analysis. InertialLAB is powered by a standard 2000 mAh power-bank. It ensures autonomy for at least eight hours considering that the InertialLAB system consumption reaches up to 120 mAh. Both the storage and power-supply unit can be easily replaced for a higher-memory or high-powered system when needed.

**Figure 2:**
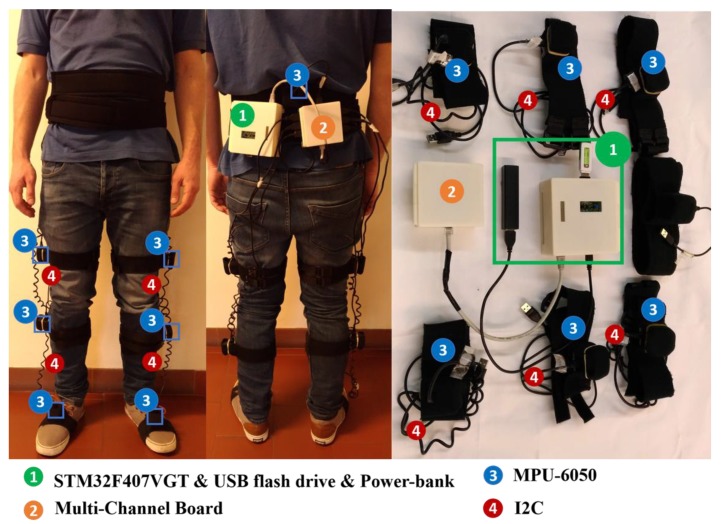
Representation and human-body location of the hardware architecture of InertialLAB.

Each IMU and the processing unit and multi-channel boards were fixed in 3D printed boxes and attached to the human using adjustable straps (Figure 2) aiming for easy usability and portability. This approach also minimizes the sensor’s relative motion to the human’s segments to avoid fluctuations in the IMU measures. The USB cables used in I^2^C communication are in the spiral form to meet the anthropometry requirements of the 10th-to-90th percentile of the male/female population (height ranging from 1.50 m to 1.90 m and weight ranging from 45 kg to 100 kg). The spiral form of the used USB cables in I^2^C communication confers this flexibility without requiring set up modifications. Further, the strap system was designed to enable good USB cables’ usability.

Figure 2 presents the hardware architecture of InertialLAB and its human-body position. The InertialLAB could cost around 242 €. This cost analysis considers the material resources (such as MPU-6050, STM32F407VGT, USB cables, mini ethernet cable, power-bank, multiplexer, and USB flash drive) and manufacturing costs of the printed circuit boards, 3D printed boxes and neoprene-made straps. The cost may increase in a future InertialLAB update with wireless technology.

### 2.3. InertialLAB: Software-In-The-Loop

InertialLAB’s software was designed to be modular and open-architecture with the possibility of full customization to operate as a stand-alone solution for general human motion analysis [14] and to be easily and directly integrated into third-party systems, namely a powered orthosis [16]. Such modularity will enable a prompt integration of the software routines into other CPU, limiting the changes to the peripheral devices’ configuration routine. Appendix A presents an instance of InertialLAB integration into a powered exoskeleton for personalized gait therapies [15].

A light-based visual feedback system was developed using the LEDs available in the CPU to inform the user about the InertialLAB’s operating status. The green LED is only turned on during the calibration routine. The blue LED turns on when the program is acquiring new data and saving it to a USB flash drive. The activation of the red LED warns the user for the occurrence of hardware (e.g., low battery, no IMU data receive, cable disconnection) or software failures (e.g., data storage error, data storage overflow) during data recording.

We implemented all software routines in STM32F407VGT. The focus was given to the IMUs’ calibration in the first 10 s of each trial and angle estimation every 5 ms (for the maximum sampling frequency of 200 Hz). Additionally, this section also describes the application of machine learning-based regression models to improve the joint angle estimation of InertialLAB with minimal drift bias.

#### 2.3.1. Calibration

**Figure 3:**
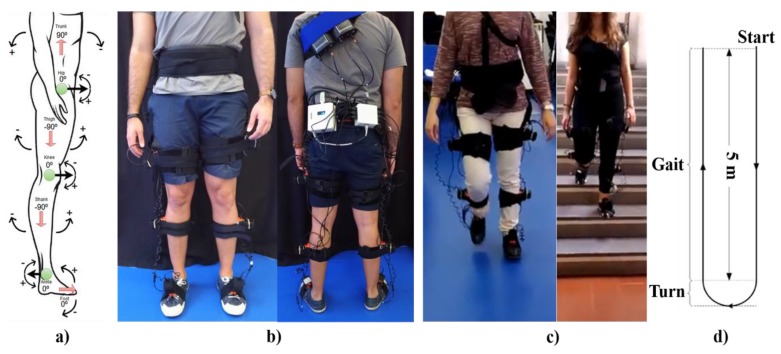
(**a**) The orientation of the segment (red arrow and the associated numbers) and joint angles (green circles and the associated numbers) in the stand-up steady-state and direction of the joint rotation (+ means the angle increases and − means the angle decreases). (**b**) Usability of InertialLAB (black boxes) and MVN BIOMECH (orange boxes); (**c**) Ongoing gait trials in flat terrain and staircase; (**d**) Turns set-up. Participants gave their informed consent to appear in the manuscript.

We implemented an automatic, user-independent, on-body calibration routine with minimum-to-null physical and cognitive effort for the user. This calibration takes place on the first 10 s of each trial, simultaneously for all IMUs, while the user is wearing the IMUs in the stand-up steady-state on a plane surface. The user should stand still in the static position with feet parallel for all 10 s, as illustrated in Figure 3a, to achieve a successful calibration. InertialLAB requires the accomplishment of the calibration procedure at the beginning of each trial for new data collection, even for the same user, and when any IMU displacement is observed. In long-term data recordings, the calibration is periodically performed every 1 h. The researcher was able to activate the calibration routine every time as needed by pressing the reset button of STM32F407VGT.

For the gyroscope’s calibration, we first computed the offset per axis ($Gyro_{offset}$) as the mean of raw gyroscope values acquired for the 10 s-calibration period. Equation (1) represents the used method to calculate the gyroscope’s calibrated value ($Gyro_{calibrated}$) for each new sample (*Gyro_raw_*), considering the computed offset (*Gyro_offset_*) and the gyroscope’s scale factor (*Gyro_scale_factor_*) set to 0.06 (as a result of the gyroscope full-scale range of ±2000°/s).

(1) $$Gyro_{calibrated}=\left(Gyro_{raw}*Gyro_{scale\_factor}\;\right)-Gyro_{offset}$$

Regarding the accelerometer’s calibration, we first computed the norm of the acceleration vector ($\left\|\vec{ACC}\right\|$), as indicated in Equation (2a), considering the mean raw acceleration measures of each axis for the 10 s-calibration period ($\bar{A_{x}\;}$, $\bar{A_{y}\;}$, $\bar{A_{z}\;}$) [22]. Subsequently, we calibrated the acceleration (*ACC_calibrated_*) through Equation (2b), where each new acceleration sample ($ACC_{raw}$ was normalized considering the accelerometer’s scale factor ($ACC_{scale\_factor}$ set to 0.00024 given the selected accelerometer full-scale range of ±8 g) and using the positive and negative component of the norm value ($\left\|\vec{ACC}\right\|$) as the maximum and minimum values for the normalization, respectively. Every time that the InertialLAB is used for kinematic gait analysis, the $Gyro_{offset}$ and $\left\|\vec{ACC}\right\|$ are initialized at zero and are automatically updated upon the first 10 s according to raw gyroscope and acceleration values, respectively, tracked in real-time during the calibration period. This calibration procedure does not apply pre-defined calibration parameters as all correction parameters are automatically determined every experiment except for scaling factors.

(2a) $$\|\vec{ACC}\|=\sqrt{\bar{ACC_{x}}{\;}^{2}+\bar{ACC_{y}}{\;}^{2}+\bar{ACC_{z}}{\;}^{2}}$$

(2b) $$ACC_{calibrated}=\frac{ACC_{raw}}{\|\vec{ACC}\|*ACC_{scale\_factor}}$$

#### 2.3.2. Angle Estimation

The angle estimation covers the real-time estimation of the lower limb segment orientation and joint angle in the sagittal plane, given its major relevance for gait analysis.

For computing the segment orientation, we explored inertial data fusion-based methods, namely the complementary filter and Kalman filter [23]. We initialized the segment orientation estimation using the trigonometry-based accelerometer method [24] since the angular velocity integration-based method cannot estimate the initial sensor orientation.

A complementary filter was implemented due to its low computational load [9]. We set 0.98 and 0.02 as the gains of the gyroscope and accelerometer contribution, respectively. These gains were found by an empiric trial-error procedure. It considered a tradeoff of the short-term reliability of gyroscope-based estimation and long-term reliability of accelerometer to minimize the drift that would arise from an entire contribution to the gyroscope.

On the other hand, the Kalman filter is more complex, more computationally expensive than the complementary filter [23]. However, it was explored, given its effective response. After a parameter tuning, we implemented the Kalman filter using the noise covariance matrix $Q_{k}$ and the measurement covariance matrix R as described in Equation (3).

(3a) $$Q_{k}=\left[\begin{array}{cc} 0.005 & 0\\ 0 & 0.0003 \end{array}\right]$$

(3b) $$R=\left[\begin{array}{cc} 0.0669 & 0\\ 0 & 0.039 \end{array}\right]$$

For joint angle estimation, we implement a trigonometry-based method dependent on the segments’ orientation values ($\theta_{Trunk}$, $\theta_{Thigh}$, $\theta_{Shank},\theta_{Foot}$)) and the assumption that in the stand-up steady-state (initial instant of gait trial) the segment and joint orientations are as described in Figure 3a. Additionally, we considered that leg segment angles vary from [−270; 180]°, the other segments vary from [−180; 180]°, and the joint angles vary from [−180; 180]°. Taking these aspects into consideration, we estimated the hip ($\theta_{Hip}$), knee ($\theta_{Knee}$), and ankle ($\theta_{Ankle}$) angles using the formulas described in Equation (4).

(4) $$\begin{array}{c} \theta_{Ankle}\left({}^{\circ}\right)=-90-\theta_{Shank}+\theta_{Foot}\\ \theta_{Knee}\left({}^{\circ}\right)=\theta_{Thigh}-\theta_{Shank}\\ \theta_{Hip}\left({}^{\circ}\right)=-\left(\theta_{Trunk}-\theta_{Thigh}-180\right) \end{array}$$

#### 2.3.3. Joint Angle Correction Using Regression Models

We explored the accuracy of non-linear regression models for correcting the InertialLAB’s joint angles using the MVN BIOMECH’s joint angles as the target measurements. The idea is to identify a well-fitted model per joint for improving the joint angle estimations of InertialLAB to minimize the drift errors. For this purpose, we trained, tuned and validated different supervised machine learning regression models based on NN [25], decision tree, and SVM [26]. We used the normalized root mean square error (NRMSE) and the coefficient of determination (R^2^) as performance metrics of 5-fold cross-validation, and Bland-Altman plots to assess the effect of regression models for drift error reduction.

The regression models were trained and tuned as follows. First, a two-layer shallow NN was trained using the Levenberg–Marquardt backpropagation algorithm [27] that considers the descend gradient of the mean of squared error (MSE). We conducted an empiric analysis to select the number of neurons in the hidden layer, varying from 5 to 20 neurons. We used a sigmoid transfer function in the hidden layer and a linear transfer function in the output layer (set with one neuron). The training stopped when any of these conditions occurred: (i) the validation error increased for 10 iterations; (ii) the MSE is minimized to zero; (iii) the performance gradient falls below $1\times 10^{-7}$; or (iv) the momentum update (*mu*) exceeds $1\times 10^{10}$.

Second, we explored the response of fine, medium, and coarse regression trees following the binary split approach [28]. The *allsplits* algorithm was used to select the best split predictor that maximizes the MSE reduction (split criterion) to decide which branch to follow. To control the tree-depth, we explored the minimum number of leaf node observations, ranging from a minimum 4, 12, and 36 observations for fine, medium, and coarse trees, respectively, to 50 and 100 observations. Additionally, we set 200 observations as the minimum number of branch node observations. Third, we investigated non-linear SVM regression models using the linear, quadratic, cubic, and Gaussian kernels with the sequential minimal optimization method [29]. Additionally, we conducted a heuristic procedure to select the C hyperparameter (box constraint), the kernel scale of the Gaussian kernel (when varying σ={0.35;1.35;2.35}), and the error margin (ε) for each input-target correspondence.

Furthermore, we investigated the effect of including the joint angular velocity as an additional input variable to the regression models since it can complement the model with dynamic data. We normalized the input variables (joint angle and joint angular velocity) and the target variables (MVN BIOMECH’s joint angles) using the min-max method within [−1; 1]. Note that the joint-dependent models were trained to be user-independent and generalized to speed variations by including in the training conditions gait cycles from different subjects walking at slow, normal, and fast self-selected speeds.

## 3. Experimental Evaluation

For the benchmarking analysis, we involved the lower-body configuration of the gold standard commercial wearable inertial system, the MVN BIOMECH [18], for three-fold reasons. First, it is a well-established wearable inertial system able to track all kinematic data monitored by the InertialLAB [1,12]. Second, the motion IMUs of Xsens demonstrated higher accuracy when compared to other commercially available IMU-based systems, such as APDM Opal and Inertial Labs OSv3, in a benchmarking analysis with Optotrak motion tracking system [30]. These findings reinforce the reliability of MVN BIOMECH as a ground truth IMU-based sensor system. Third, the MVN BIOMECH is a wearable solution able to track the human gait in ambulatory scenarios like those explored in this work. We did not consider the use of camera-based motion systems since they do not provide benchmarked measures for the angular velocity and acceleration and do not allow an ambulatory gait analysis in non-structured and outdoor environments. Additionally, the literature’s results report that MVN BIOMECH is a valid tool for quantifying kinematics during functional movements when compared to camera-based motion systems. The orientation information provided by an IMU had shown to be accurate [8,30]. The lower-limb joint angles provided by MVN BIOMECH software in the sagittal plane were scored with an excellent validity and fair-to-excellent reliability for healthy participants performing overground walking [31,32] and for climbing stairs [32].

We centered the benchmarking analysis to the kinematic data monitored by InertialLAB to evaluate its operability for gait analysis. However, the MVN BIOMECH includes further gait analysis functions, and it can perform a full 3D tracking of the body motion using wearable IMUs (MTw Awinda) and native biomechanical tools.

### 3.1. Participants

We included 11 able-bodied subjects (7 males and 4 females). The participants’ mean age was 24.53 ± 2.09 years old, with a height of 1.71 ± 0.10 m and a body mass of 65.29 ± 9.02 kg. All participants provided written informed consent to participate in this study. The study was conducted according to the rules of ethical conduct of the Life and Health Sciences defined by the University of Minho Ethics Committee, addressing the principles of the Declaration of Helsinki and the Oviedo Convention.

### 3.2. Protocol and Data Collection

The participants wore their sport-shoes and 7 IMUs in the configuration depicted in Figure 3b. To ensure the repeatability of the sensor’s alignment in the leg, the assessor identified and marked the lateral side in the middle of the thigh and shank segments [33]. For the trunk and foot segments, the assessor identified the lower back position (near to the center of mass) aligned with the spinal cord and the instep position aligned with the navicular bone, respectively. The sensors of InertialLAB and MVN BIOMECH were placed on these positions by the assessor, who used the double holder straps of InertialLAB to ensure that its sensors are aligned and fixed over the IMUs of MVN BIOMECH, as shown in Figure 3b. We used a hardware-based sync method (TTL sync) to synchronize both systems.

The MVN BIOMECH performance may be affected by base station distance. Consequently, before starting the experiments, the assessor strategically studied the best position of the base station to ensure adequate wireless communication from the IMUs of MVN BIOMECH to the base station during all data acquisition period. The base station position in the real-world scenario was established where the MVN Analyse software, already validated by the Xsens, rated a good wireless communication signal.

Each trial started by calibrating the MVN BIOMECH in N-pose considering anthropometric user data (height and foot length). The experiments only proceeded after the successful finalization of the calibration procedures in the intended walking environment. Subsequently, we asked the participant to stay in the stand-up steady-state in N-pose for 10 s to calibrate InertialLAB, and then, he/she could start the gait trial. The data were collected and stored for a posteriori analysis at 100 Hz, the maximum sample rate allowed by MVN BIOMECH.

The participants were asked to randomly perform 9 trials at three self-selected gait speeds (slow, normal, and fast) on a10 m-flat surface. Additionally, the subjects randomly conducted 10 gait trials on two real-world terrains (Figure 3c), at a self-selected gait speed. In the first terrain, they walked 2 m forward in level-ground; ascended a staircase; walked forward in level-ground for 2 m and stopped; turned around and descended the staircase back to the starting position. The indoor staircase had 8 steps with 17 cm of height, 31 cm of depth, and 110 cm width. On the second circuit, the participants walked 2 m forward in level-ground; ascended a ramp; walked forward in for 2 m and stopped; turned around and descended the ramp back to the starting position. The outdoor ramp was 10 m with a 10° inclination.

Furthermore, we asked the participants to conduct trials with turns (Figure 3d) to assess the increment of the InertialLAB’s drift error in this performed daily-activity. Each participant performed 9 trials as follows: walked forward for 5 m, changed the walking direction with a 180° turn, and walked forward to back to the starting position.

Additionally, we studied the InertialLAB’s usability collecting the end-users’ perception and satisfaction upon the InertialLAB’s use in daily locomotion conditions. At the end of the experiment, each participant was asked to fulfill the System Usability Scale (SUS) questionnaire available as a paper-and-pencil tool. No time constraints were established, such that the subjects had enough time to complete the questionnaire. The participants were queried regarding the InertialLAB’s usability through a 10-item questionnaire proposed in [19] that uses the 5-point Likert scale. The SUS is a well-known usability questionnaire since it is a reliable, stand-alone evaluation valid to be applied to any product [34]. Consequently, it was involved in InertialLAB’s usability assessment. All questionnaires were collected for posterior analysis.

### 3.3. Data Analysis

We used the Matlab^®^ (2017b, The Mathworks, Natick, MA, USA) for the benchmarking analysis of 3D angular velocity, 3D acceleration, and joint angles as follows, without considering the acceleration and deacceleration zone. First, we computed the NRMSE and metrics for assessing the waveform similarity, such as the correlation coefficient (ρ) and cross-approximate entropy (XApEn [35]). These analyses were conducted separately for every sensor unit and trial varying gait speed and terrain to evaluate the operability of IntertialLAB considering the sensor’s location, gait speed, and the terrain.

Second, we assessed the drift error in the joint angle estimations to investigate the effectiveness of the proposed trigonometry-based method upon to inertial data-based fusion methods comparatively to the biomechanical model-based approach of the MVN BIOMECH. As such, we computed the ratio among the InertialLAB’ drift error and the MVN BIOMECH’s drift error. For both sensor systems, we computed the drift error as the slope of the linear trend of the joint angle signals (by assuming that the drift error follows a linear trend throughout the gait trial [5]). For trials including turns, we determined the percentage of the increment of drift with the 180° turn by comparing the drift error before and after the turning. Third, we conducted a statistical analysis to investigate the hypothesis that NRMSE comes from a distribution with mean zero using the one-sample *t*-test with a significance level of 5%.

Lastly, we performed InertialLAB’s usability assessment. The mean SUS score and InertialLAB’s usability grade were determined considering the eleven answered questionnaires. The SUS score was computed according to the questionnaire guidelines [19], as follows: (i) for odd items (1, 3, 5, 7, and 9), we subtracted one from the user response to get the score contributions; (ii) for even-numbered items (2, 4, 6, 8, and 10), the contribution is 5 minus the user response; (iii) we added up the score contributions for each user and multiply by 2.5 to obtain the SUS score (range from 0 to 100); (iv) SUS scores obtained from eleven questionnaires were averaged to determine the overall value of SUS score for InertialLAB. Additionally, we analyzed the distribution of the users’ reports to each item of the questionnaire through a histogram in an attempt to verify the need for additional usability improvement.

## 4. Results

We observed technical remarks during the experiments as follows. InertialLAB’s calibration requires a minimum-to-null physical and cognitive effort for the users and assessor. MVN BIOMECH’s performance was highly dependent on the calibration’s environment and the distance to the base station. The power supply system of MVN BIOMECH was replaced every 60 min while no charging periods for the InertialLAB’s power supply system needed along with daily consecutive recording sessions. Moreover, from the optical-modeling tools of an open-source tracker, we verified a comparable performance between the complementary filter and Kalman filter (differences were lower than 0.2° with RMSE < 6.5°) for the segment orientation. The complementary filter was used for segments’ orientation estimation, given the similar performance of both fusion methods and the lower computation load of the complementary filter. The InertialLAB software routines executed with a mean computation time of 2.4 ± 0.47 ms, with 95% of the samples being computed within 3.1 ms for a CPU running at 168 MHz.

### 4.1. Benchmarking Analysis

The benchmarking analysis centered on the kinematic data monitored by InertialLAB (3D-angular velocity, 3D-acceleration, and sagittal joint angles) to evaluate its operability at three self-selected gait speeds (slow: 0.83 ± 0.11 m/s, normal: 1.09 ± 0.16 m/s, and fast: 1.59 ± 0.17 m/s) throughout three non-structured terrains (flat, staircase, and ramp).

Table 1 shows the mean values of NRMSE, ρ, and XApEn for 3D-angular velocity. The error of the gyroscope embedded on the InertialLAB tends to increase with the gait speed (mean NRMSE varied from 0.08 to 0.104 as speed increases). On the other hand, the InertialLAB’s angular velocity signals become more similar (ρ increases) and synchronous (XApEn decreases) to ones of MVN BIOMECH as the speed increases. Comparing with MVN BIOMECH, the gyroscope of the InertialLAB performed better in stair ascend and descend as indicated by the lower magnitude-error and higher waveform similarity.

In overall, the acceleration signals of the InertialLAB showed higher magnitude-errors (NRMSE > 0.09) and fewer signals’ correlation (ρ < 0.768) than the ones found in the gyroscope. Table 2 shows that the acceleration signals of InertialLAB presented a similar performance in magnitude (0.114 < NRMSE < 0.117) and waveform correlation (0.721 < ρ < 0.73) when the gait speed varies, and were more robust for the stairs ascent and descent activities, as indicated by the lower magnitude-error (NRMSE = 0.09 ± 0.021) and the higher waveform similarity (ρ = 0.768 ± 0.037).

The findings stated in Table 3 indicated that the waveform similarity between the InertialLAB’s joint angles and MVN BIOMECH’s joint angles increases as the speed increases (ρ increases from 0.899 to 0.909 whereas XApEn reduces from 0.082 to 0.075). On the other hand, the NRMSE increases with the gait speed. Moreover, the joint angle estimations were better in-magnitude in indoor flat surfaces (the lowest NRMSE) than an outdoor ramp and indoor staircases. However, the joint angle signals of the InertialLAB tracked in ramp and staircases tend to be more correlated (increment of the mean ρ from 0.898 to 0.944) and less dissimilar (reduction of mean XApEn from 0.082 to 0.051) with the paired joint angles of MVN BIOMECH for these terrains. From the statistical analysis, we verified that the mean NRMSE values found for the angular velocity, acceleration, and joint angle are significantly different from zero (*p* < 0.05).

**Figure 4:**
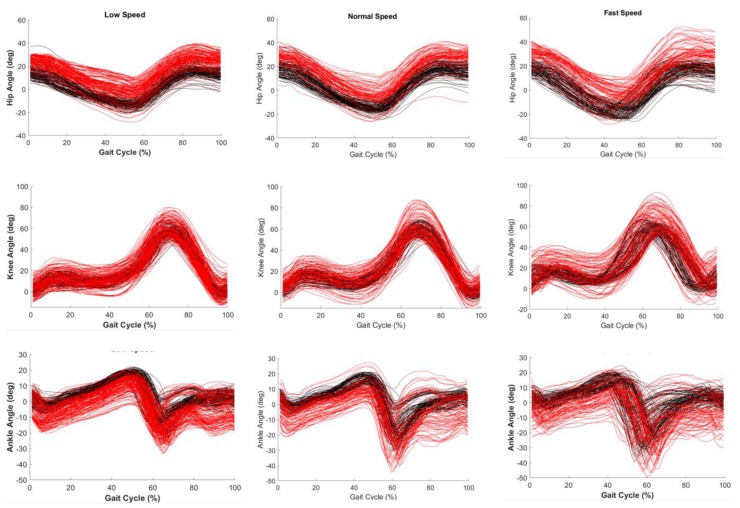
Hip (**top row**), knee (**middle row**) and ankle (**bottom row**) angles in the sagittal plane of some strides for all subjects wearing the InertialLAB (red) and MVN BIOMECH (black) throughout the gait cycle at low (1st column), normal (2nd column), and fast (3rd column) speed in flat terrain.

**Figure 5:**
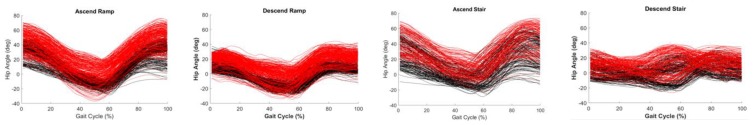

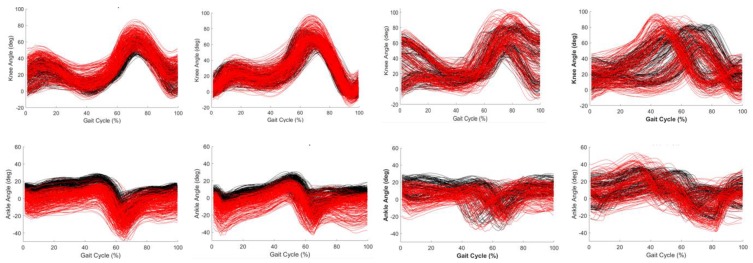
Hip (**top row**), knee (**middle row**) and ankle (**bottom row**) angles in the sagittal plane of some strides for all subjects wearing the InertialLAB (red) and MVN BIOMECH (black) throughout the gait cycle for ascend ramp (1st column), descend ramp (2nd column), ascend stair (3rd column), and descend stair (4th column).

Furthermore, we compared the joint angle time-series (as presented in Figure 4 and Figure 5) of InertialLAB and MVN BIOMECH given the relevance of joint angles for the InertialLAB’s application in the gait analysis. Results of Figure 4 show that the magnitude-errors between both sensor systems tend to augment as the gait speed increases, as verified in Table 3. Additionally, the speed increment leads to an increased range of motion, mainly observed for ankle joint.

By analyzing Figure 4 and Figure 5, it is observed that the range of motion of the lower limb joints varies according to the terrain, mainly for hip and ankle joints. Higher differences were found in ramp and stair ascent. Figure 5 reports a dissimilar waveform throughout the gait cycle, suggesting that there is a divergent gait pattern between the included participants when walking in the staircase. Across the terrains, there appears to be a good similarity between the pattern of sagittal joint angles monitored by both systems, as indicated by the results of Table 3. The results of Figure 4 and Figure 5 show an offset in the joint angles, especially for the ankle joint, resulting from the drift error and other systematic errors. Table 4 presents the mean ratio of drift error of InertialLAB and MVN BIOMECH, per lower limb joint, speed, and terrain. Overall, the InertialLAB’s drift error was more pronounced in the ankle joint and less observed in the knee and tends to increase with the gait speed (as demonstrated in Figure 4 and Figure 5). The computed ratio of drift error was higher when walking in a ramp, mainly for the ramp descent, but comparable for walking in flat terrain and staircase. Moreover, Table 4 shows the mean percentage of the drift error increment with 180° turns. The findings indicate that turns affect the joint angle estimations of both systems, but with double effect in the InertialLAB, the ankle being the joint most sensitive to the turns.

**Figure 6:**
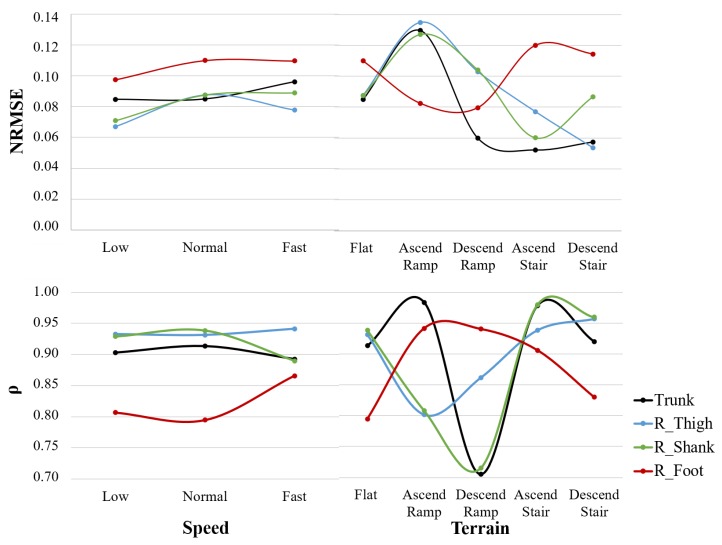
Evolution of NRMSE and ρ for the angular velocity throughout the self-selected speed and terrain.

Lastly, we compared the evolution of the similarity (NRMSE and ρ) between the InertialLAB and the MVN BIOMECH per sensor unit to identify the less efficient IMU’s positioning of the InertialLAB. As an instance, Figure 6 presents the evolution of NRMSE and ρ for angular velocity considering the IMUs placed on the trunk, and right thigh, skank, and foot. From this analysis, we verified that the IMU placed on the foot was the less effective sensing unit in terms of magnitude (higher NRMSE) and waveform similarity (lower ρ). These results are consistent as the speed increases. Indeed, the results of Figure 6 confirm the trend for increasing NRMSE with gait speed and a higher error in the ramp when compared to other terrains, as analyzed in Table 1. Moreover, the performance of the IMU placed on the foot was divergent from the other IMUs for the studied terrains; it showed a smaller error and higher waveform similarity in ramp, whereas the performance of the remaining IMUs was degraded in this condition and well-performed in stairs. Additionally, Figure 6 demonstrates that the representative increment of ρ for foot sensor may explain how the InertialLAB’s angular velocity signals become more similar to ones of MVN BIOMECH as the speed increases.

### 4.2. Regression Models

The regression models were assessed with 31,500 observations recorded from all participants (11 able-bodied subjects) for three self-selected walking speeds (slow, normal, and fast speeds), considering the protocol above described. It was observed that the performance of all regression models improved with the inclusion of the joint angular velocity, rather than the single-use of the joint angle as input. Overall, the prediction error reduced 13% with the inclusion of joint angular velocity. Table 5 depicts the regression models with higher performance. The regression models tuned for the knee joint were more robust (NRMSE < 0.069, R^2^ > 0.90), whereas the ankle-based regression models provided less accurate angular predictions (NRMSE < 0.092, R^2^ > 0.69).

Overall, the shallow NN with 5 neurons in the hidden layer showed to be the better-fitted regression model to predict the hip, knee, and ankle angle (R^2^ = 0.92, R^2^ = 0.94, and R^2^ = 0.87, respectively). The comparative analysis showed that the SVM-based linear and quadratic kernels, and the fine and medium trees were less accurate than the models in Table 5. It is possible to note that the speed-independent regression models, particularly the NN regression model, reduced the magnitude-based errors in the hip, knee, and ankle angle estimated by the InertialLAB. This remark may be explained by the similar NRMSE values in Table 5 (0.069 < NRMSE < 0.092) and Table 3 (0.066 < NRMSE < 0.07) and considering that the results of Table 3 did not reflect the drift-dependent errors, which were expressive as listed in Table 4.

**Figure 7:**
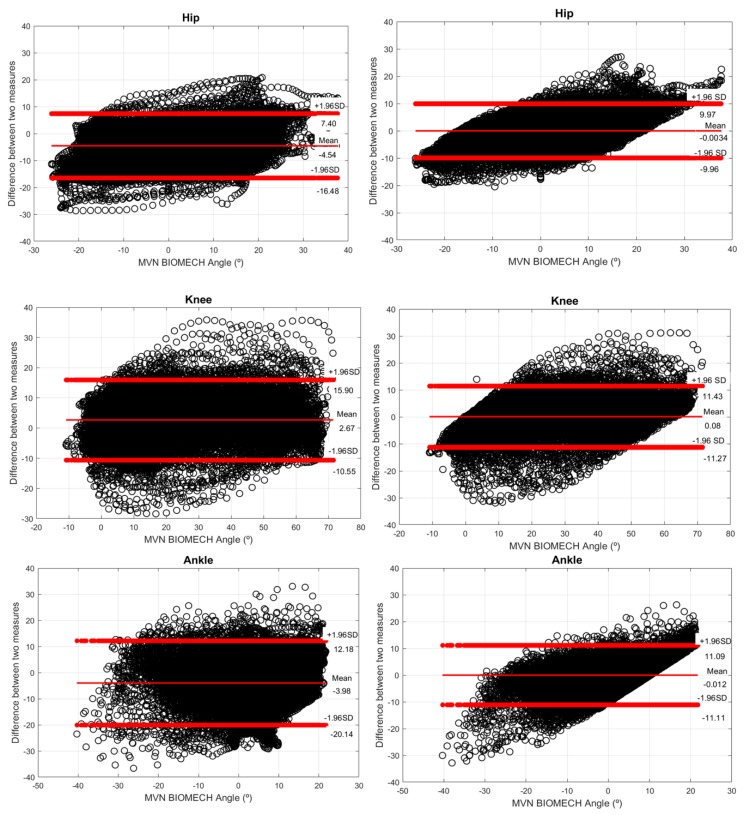
Bland-Altman plots of InertialLAB’ joint angle estimations (**left view**) and the joint angle predictions by the NN (**right view**) against the angles of MVN BIOMECH for different self-selected walking speeds (slow, normal, and fast speeds). The red horizontal lines represent the mean difference and the 95% limits of agreement (i.e., mean difference ± 1.96 SD of the difference).

Additionally, to evaluate the accuracy of the NN regression model in the joint angles’ improvement, we compared the agreement between the NN predictions and the joint angles estimated by MVN BIOMECH. The Bland–Altman plots illustrated in the left view of Figure 7 indicate the presence of bias in the joint angles estimated from fusion-based methods given the non-zero mean difference values (−4.54, 2.67, and −3.98 for the hip, knee, and ankle, respectively). In opposition, the mean difference is closer to 0° after the NN regression model application (right view of Figure 7), suggesting that the bias, such as the drift error, is approximately null for the NN’s joint angle predictions. Figure 7 also shows a reduction in the limits of the agreement after the application of the NN regression model. The reduction was more pronounced for the ankle regression model that varied from [20.14; 12.18]° to [−11.11; 11.09]° and less expressive for the hip models, ranging from [−15.48; 7.40]° to [−9.96; 9.97]°. This finding is explained by the higher drift errors observed in the ankle joint angles when compared to the other joints, resulting in a wider margin for NN corrective effects. Moreover, Figure 7 illustrates that the improvements introduced by the NN model are more expressive in the middle of the joints’ range of motion, as higher differences in the joint angles of both systems are observed at both limits of the range of motion.

### 4.3. InertialLAB Usability Assessment

**Figure 8:**
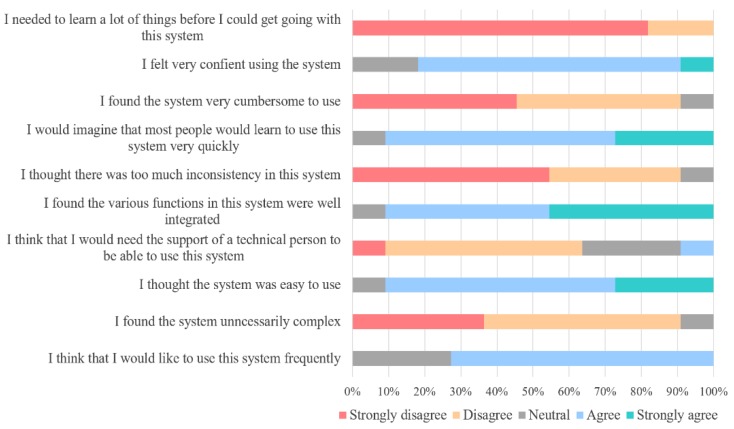
Percentage distribution of the score given by participants to the 10-item system usability scale.

The InertialLAB’s usability reached a mean SUS score of 79.77, corresponding to A^−^ grade. This result indicates that the users consider that the InertialLAB presents good usability [19]. Figure 8 shows a global distribution of the users’ satisfaction for each queried item. Most of the participants agreed that: they would like to use the InertialLAB frequently (72.7%); the system was easy to use (63.6%); they felt very confident using the system (72.7%); and, they would imagine that most people would learn to use this system very quickly (63.6%). Further, they strongly disagree that there is too much inconsistency in this system (54.5%), the system is very cumbersome to use (45.5%), and that they needed to learn a lot of things before using the system (81.8%).

## 5. Discussion

### 5.1. System Design and Usability and Reliability Analysis

This study introduced a cost-effective, low-power consumption, and easily calibrated wearable inertial sensor system. It was designed for monitoring 3D angular velocity and 3D acceleration up to 6 lower limb segments and trunk and joint angle in the sagittal plane up to 6 joints. The main contributions of InertialLAB design are as follows. First, the use of an automatic, user-independent, on-body calibration routine in the first 10 s of the data monitoring avoids higher time-consuming calibration methods with several manual maneuvers per IMU, as proposed in [5,6]. Moreover, the approached calibration routine is generic to any user and demands minimum-to-null physical and cognitive effort for the user and assessor, which favors its daily application.

Second, the InertialLAB design centered on a multiplatform insight, considering both stand-alone and system-cooperative functioning. The software followed a modular and open-architecture to be easy, time-effectively, and directly integrated into third-party systems, advancing the commercial IMU-based systems, for monitoring kinematic data up to 200 Hz with minimal latency. Additionally, the hardware design of the power supply and storage unit of InertialLAB also showed a modular character by enabling their easy replacement as needed. Third, the power supply unit of InertialLAB showed to be advantageous than the one used in MVN BIOMECH regarding the durability and usability (power unit of 20 × 20 × 100 mm vs. two power units of 60 × 50 × 150 mm). Furthermore, it includes software interfaces operating up to 200 Hz with a low computational load to enable its execution into a wearable CPU (80 × 100 × 25 mm) when compared to MVN BIOMECH (higher-dimensionality CPU such as a personal computer) and the one (200 × 137 × 55 mm) used in [6]. This allows a more practical application of InertialLAB for the ambulatory gait analysis. The findings also indicate that the selected wearable CCU is able to timely execute the software routines of InertialLAB (the mean computation time of 2.4 ± 0.47 ms is lower than the timing requirements of 5 ms) that were implemented for real-time data acquisition and processing towards kinematic gait data analysis. There is still room to include further computational methods into InertialLAB.

Additionally, this work goes forward to related studies, which addressed the inertial system validations [4,5,6,11,13], by performing a usability assessment. The users report that InertialLAB presents good usability after their experience when interacting with this sensor system in daily conditions. Its good usability is an important quality attribute for suggesting the use of InertialLAB in daily kinematic gait analysis.

Standard methods to ensure the system reliability and reduced the probability to failure were followed: (i) use charged power-bank at the beginning of each experimental procedure; (ii) employment of robust USB cables to reduce the probability to failure; (iii) development of overall hardware into a custom-designed printed circuit board; (iv) use of robust straps to ensure proper and lasted IMUs attachment to the human body, avoiding displacements; (v) reduce system vibration by fixing all hardware modules in custom-designed 3D printed boxes through M2 screws; (vi) release USB flash drive memory at the end of each experimental procedure for a remote database. Additionally, we ensure consistency in the calibration procedure since the user should remain in stand-up steady-state while the green LED is on (corresponds to 10-s calibration routine), and the subject can only start walking as the calibration is completed, i.e., when the blue LED is turned on. Software issues were solved during the systematic technical validation testes that were performed prior to data collection with end-users. However, the InertialLAB reliability may be compromised by occasional failures, such as USB cable disconnection stop IMU data collection, data storage error due to USB flash driver disconnection from CPU, and data storage overflow. Additionally, the quality of data acquisition depends on an IMU body placement, which should be laterally placed over bone zones.

### 5.2. Kinematic Gait Analysis

The implications of the sensor technology and kinematic gait analysis of InertialLAB are discussed below by considering the benchmarking analysis against the MVN BIOMECH. Variations in gait speed may affect the performance of the gyroscope and accelerometer embedded in the InertialLAB by increasing the magnitude-based errors. This may be explained by the increment of the IMUs’ attachment instability as speed increases. There is evidence the waveform similarity of angular velocity signals improves as the gait speed increases. The fluctuations in the gyroscope performance may result from its dependency on the temperature [9] since the experiments occurred indoor and outdoor at different times. The MPU-6050 was selected due to its low latency performance and its widespread use for developing low-power and low-cost motion tracking devices [19]. However, the MPU-6050 is susceptible to temperature changes and requires careful calibration in order to make it useable. Despite caution during calibration, the findings may suggest the need for technological improvements in MPU-6050 when compared to IMU of MVN BIOMECH.

Furthermore, the performance of the IMUs embedded in the InertialLAB was better in climbing stairs than in flat terrain and ramp. Two reasons may explain this. First, it may result from a shorter walked distance in the staircase comparing to other terrains. Second, climbing stairs may deal with a slight impact interaction with the ground, resulting in lower secondary motions of the IMUs than in other terrains. To the best knowledge of the authors, there is no study in the state-of-the-art exploring the reliability of the gyroscope and accelerometer in a wearable inertial sensor system for gait analysis in terrains commonly encountered in daily routine. The sensors’ comparative analysis in these conditions is itself a contribution to the state-of-the-art.

The proposed joint angle estimation method is fairly simple. It is important to stress that a central requirement in our system was to achieve a low computational load (95% of the samples were computed within 3.1 ms) in wearable CPU towards ambulatory human motion analysis and for interfacing with wearable assistive devices. Comparing to concurrent wearable systems, InertialLAB does not involve manual measurements [11] nor kinematic constraints assuming the symmetry of the limbs [8]. The fusion method, followed by a trigonometry-based approach, estimated the joint angles with a high waveform similarity (as shown in Table 3) and time-series regularity (as illustrated in Figure 4 and Figure 5) when compared with MVN BIOMECH. The waveform similarity was more evident for climbing stairs, which is in line with previous reports. The studies [31,32] reported an excellent waveform similarity (>0.9) for the three joints measured by MVN BIOMECH system for walking [31,32], jumping activity [31] and climbing stairs [32]. Tadano et al. [11] have shown high correlations (ρ > 0.78) for three joints in a 5-m flat surface. The InertialLAB presented a comparable performance in a 10 m-flat surface (mean ρ = 0.905 for three joints). According to [11] and [31], the high correlation in joint angle time-series (ρ > 0.898 for the three terrains) can be interpreted as high reliability and excellent validity of the InertialLAB, respectively.

Nonetheless, there is significant evidence (*p* < 0.05) for the discrepancy on the joint angles in the form of offset, such as the drift error, as reported in Figure 4 and Figure 5. The offset-based errors in the InertialLAB’s joint angle estimations tend to increase with the gait speed and were more expressive for descending ramp likely due to the occurrence of higher oscillatory artifacts in the suspended ramp or higher environment influences in this outdoor scenario. Previous works also reported offset-based errors for the lower limb joint angles estimation when using the quaternion algorithm upon the Kalman filter [5,6,11]. Liu et al. [5] reported a maximum RMSE of 16.6° along with trials with 3 strides; Beravs et al. [6] reported a mean error lower than 5° when one subject walked 30 steps; and, Tadano et al. [11] found a mean RMSE ranging from 7.88° to 10.14° from a gait analysis on 5 subjects along 5 m. These works limited the validation of IMUs-based kinematic analysis to short distance gait trials with few participants. It may limit the analysis of the repeatability of the literature fusion methods, mainly when their performance tends to decrease over time. On the other hand, it could be argued that the errors reported in the InertialLAB’s validation reflect a number of heterogenous gait patterns from non-structured scenarios, approaching the repeatability performance of the system with different issues faced in daily walking.

Our results report that the drift error was more pronounced in the ankle joint, as reported in [6,11,31]. Particularly, the findings of Figure 6 report that the kinematic analysis of the IMU placed on the foot is the least similar to the equivalent sensor of the MVN BIOMECH. Often the most distal segments move the most during gait; therefore, they are more susceptible to fluctuations and signal distortions. It was also observed a low-effectiveness in magnitude and waveform regularity of the IMU located on foot likely due to the secondary motions introduced at heel-strike and toe-off timings [11]. Additionally, the fairly computational proposed approach for joint angle estimation is twice as sensible to the turns than MVN BIOMECH, which also showed to be sensitive [30].

With this study, particularly by analyzing the results of Figure 7, we verified that the NN regression models successfully attenuated the bias (decreased from 4.54° to approximately 0°) and increased the agreement between the joint angle series of InertialLAB and MVN BIOMECH. These findings suggest that the NN regression models can successfully be applied to minimize the bias in joint angle signals and to yield signals with an excellent validity (R^2^ = 0.92 for hip, R^2^ = 0.94 for knee, and R^2^ = 0.87 for ankle) [31]. Ankle-based regression models provided less accurate angular predictions, which is in line with the literature reports high incoherencies in ankle joints [6,11,31]. There is evidence that the ankle is the lower limb joint less effectively modeled. Furthermore, the outcomes demonstrated that NN ability to improve joint angle estimation benefits from the inclusion of the joint angular velocity as an additional input to the joint angle estimated by fusion methods. The joint angular velocity likely contributed to NN robustness to predict joint angles under walking speed variations. When compared to MVN BIOMECH, there is still room to improve NN regressor capabilities on bias reduction at the limits of the joint’s range of motions in multi-terrain walking.

The proposed speed-independent regression model yielded similar results to the ones reported in [36] when using a phase-locked regression model that exploits the cyclical nature of human gait. However, this literature approach depends on the effectiveness of gait cycle segmentation issued by an extra computational tool [36]. On the other hand, NN regression models are a generic approach tuned to be user-independent and generalized to speed variations aiming for a versatile application of InertialLAB in biomechanical analysis. This study innovatively demonstrates the promising use of machine learning regression models for improving the accuracy of wearable inertial sensor systems for daily kinematic gait tracking.

The hardware modular design and the software open architecture of InertialLAB enable its direct integration, with low computational load, into third-party wearable devices or computational models for timely providing real-time kinematic gait data to these systems as needed. This key feature was attained, aiming the need for a personalized clinical insight considering the user’s actual kinematic condition. The relevance of this direct integration has also been reported in literature works for a prosthesis [9] and an exoskeleton [6]. However, the validity of these sensor systems for daily kinematic gait analysis was not inspected. In light of its low computational complexity, good validity, and usability, InertialLAB shows the reliability for providing real-time kinematic data to third-party systems into daily assistive conditions.

### 5.3. Limitations and Future Directions

The application of the InertialLAB for kinematic gait analysis may face limitations, as follows. First, the differences in the real joint kinematics and the assumptions considered in the calibration may introduce a fixed bias. Second, the effectiveness of the proposed calibration is affected by incorrect postures during the stand-up steady-state. Third, the existence of sensor misalignments regarding the human joint axis, mainly in long-term recording sessions. Fourth, the wired sensors’ connection may extend the installation of the sensors on the users’ body and may not be the most practical solution for daily human gait analysis. However, likely due to the cables’ flexibility, the participants did not report constraints while using the InertialLAB at different daily locomotion modes in indoor and outdoor environments. Contrariwise, they assessed the InertialLAB with good usability.

Furthermore, the tackled benchmarking analysis may be limited by the absence of magnetometers in the InertialLAB and likely by the increased misalignments of InertialLAB’s IMUs comparing to MVN BIOMECH’s IMUs as speed increases given the positioning of InertialLAB over MVN BIOMECH. The comparison should also consider the specific development of InertialLAB for gait analysis, whereas the MVN BIOMECH approaches a full 3D motion tracking, involving more complex components than the ones in InertialLAB. This study used the validated MVN BIOMECH as a ground truth wearable IMU-based sensor system for kinematic gait analysis, without re-evaluating its validity. However, there may be minor inaccuracy in the performed benchmarking analysis due to the distance of the IMUs of MVN BIOMECH to the base station and the environment influence on calibration. Moreover, MVN BIOMECH is sensitive to the effect of the magnetic field environment. The concurrent validity of the MVN BIOMECH with camera-based motion systems reported some magnitude-based errors [31,32]. However, these results should not be considered a failure on the part of the MVN BIOMECH solely. Other commercial IMU-based sensor systems, such as RIABLO (CoReHab, Trento, Italy), G-walk (BTS Bioengineering Corp., Quincy, MA, USA), Shimmer Sensing (Dublin, Ireland), and APDM Wearable Technologies (Portland, OR, USA), could be used to further validate the effectiveness of the proposed system.

Additionally, this study investigates the hypothesis that a machine learning-based regression model may accurately improve the joint angle estimation of InertialLAB with minimal drift bias by comparing different regression models (NN, decision tree, and SVM). The regression model comparison was limited to the prediction accuracy without considering the computational efficiency.

More research is needed to improve aesthetic and functional issues in InertialLAB to integrate a wireless solution upgrading the usability. Future work includes the sensor’s misalignments mitigation and misalignment compensation between IMUs and the human joint axis. The monitoring of 3D joint angles may enable InertialLAB’s use in further applications.

## 6. Conclusions

We proposed a cost- and time-effective, low-power consumption, wearable inertial sensor system with an automatic, on-body calibration with a minimum-to-null effort for the user and assessor or therapist. The computational joint angle estimation method presented high reliability and excellent validity in the waveform while operating into non-structured scenarios. The user-independent NN regression models successfully minimized the bias in joint angle signals in varying gait speed, turns, and terrain. NN regression methods potentially eliminate the need for high-complex biomechanical models and gait cycle-dependent methods for reliable joint angle estimation in daily conditions. InertialLAB follows a modular, open-architecture with good usability, enabling (i) out-of-the-lab analysis for understanding human gait beyond constrained laboratory conditions, and (ii) real-time kinematic data translation to third-party wearable devices.

## Figures and Tables

**Table 1:** Mean NRMSE, ρ, XApEn per speed and terrain: 3D Angular Velocity.

Terrain	Speed	NRMSE	ρ	XApEn
Flat	Low	0.08 ± 0.012	0.859 ± 0.062	0.069 ± 0.017
	Normal	0.103 ± 0.017	0.863 ± 0.082	0.052 ± 0.058
	Fast	0.104 ± 0.018	0.871 ± 0.037	0.043 ± 0.079
Ramp ascend	Normal	0.117 ± 0.024	0.857 ± 0.078	0.051 ± 0.029
Ramp descend	Normal	0.103 ± 0.026	0.807 ± 0.139	0.057 ± 0.049
Stair ascend	Normal	0.082 ± 0.047	0.925 ± 0.076	0.051 ± 0.032
Stair descend	Normal	0.083 ± 0.037	0.903 ± 0.057	0.062 ± 0.026

**Table 2:** Mean NRMSE, ρ XApEn per speed and terrain: 3D Acceleration.

Terrain	Speed	NRMSE	ρ	XApEn
Flat	Low	0.117 ± 0.017	0.730 ± 0.047	0.083 ± 0.025
	Normal	0.118 ± 0.010	0.728 ± 0.058	0.081 ± 0.028
	Fast	0.114 ± 0.010	0.721 ± 0.049	0.085 ± 0.043
Ramp ascend	Normal	0.106 ± 0.014	0.726 ± 0.074	0.076 ± 0.044
Ramp descend	Normal	0.121 ± 0.013	0.730 ± 0.114	0.085 ± 0.043
Stair ascend	Normal	0.103 ± 0.044	0.764 ± 0.046	0.077 ± 0.058
Stair descend	Normal	0.090 ± 0.021	0.768 ± 0.037	0.082 ± 0.040

**Table 3:** Mean NRMSE, ρ, XApEn per speed and terrain: Sagittal Joint Angle.

Terrain ^1^	Speed	NRMSE	ρ	XApEn
Flat	Low	0.066 ± 0.01	0.898 ± 0.042	0.082 ± 0.021
	Normal	0.067 ± 0.008	0.905 ± 0.049	0.080 ± 0.014
	Fast	0.070 ± 0.009	0.909 ± 0.063	0.075 ± 0.012
Ramp ascend	Normal	0.086 ± 0.012	0.936 ± 0.08	0.051 ± 0.020
Ramp descend	Normal	0.084 ± 0.011	0.931 ± 0.035	0.053 ± 0.025
Stair ascend	Normal	0.098 ± 0.007	0.930 ± 0.013	0.057 ± 0.013
Stair descend	Normal	0.088 ± 0.004	0.944 ± 0.014	0.061 ± 0.027

^1^ NRMSE values do not reflect the drift errors of both sensor systems for performing an independent evaluation, as presented in Table 4.

**Table 4:** Drift error measures per joint, speed, and terrain.

Terrain	Speed	Hip	Knee	Ankle
Mean ratio of the InertialLAB’s drift error and MVN BIOMECH’s drift error				
Flat	Low	2.7	2.8	3.9
	Normal	3.2	2.5	4.1
	Fast	3.5	3.2	4.9
Ramp ascend	Normal	5.4	4.6	5.5
Ramp descend	Normal	6.5	5.6	6.9
Stair ascend	Normal	4.7	4.0	4.2
Stair descend	Normal	2.6	2.3	3.7
Mean % of the drift error increment after a 180° turn				
MVN BIOMECH		25.29	27.71	27.46
InertialLAB		41.44	42.99	59.72

**Table 5:** Regression models better-tuned for improving joint angles.

Joint	Regression Method	NRMSE ^1^	R^2^
Hip	Shallow NN (hidden layer with 5 neurons)	0.079	0.92
	Tree (Coarse kernel minimum leaf = 100)	0.080	0.84
	SVM (Fine (σ=0.35) and Medium (σ=0.4) Gaussian kernel, C=0.52, ε=0.052)	0.079	0.85
Knee	Shallow NN (hidden layer with 5 neurons)	0.069	0.94
	Tree (Coarse kernel minimum leaf = 100)	0.069	0.90
	SVM (Fine (σ=0.35) and Medium (σ=0.4) Gaussian kernel, C=0.23, ε=0.023)	0.069	0.90
Ankle	Shallow NN (hidden layer with 5 neurons)	0.092	0.87
	Tree (Coarse kernel minimum leaf = 100)	0.091	0.69
	SVM (Fine (σ=0.35) and Medium (σ=0.4) Gaussian kernel, C=0.23 ε=0.023)	0.092	0.69

^1^ NRMSE values reflect the drift error of both sensor systems.

**Figure A1:**
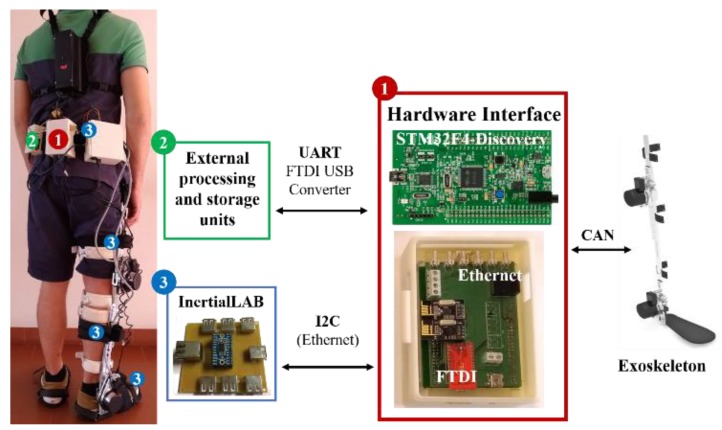
Conceptual architecture and usability of the hardware interfaces for integrating the InertialLAB into ankle and knee exoskeletons.

**Figure A2:**
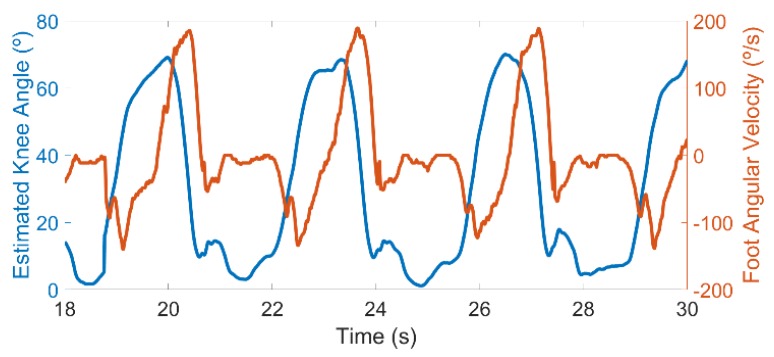
Representative results of the InertialLAB’s integration into the ankle exoskeleton.

## Appendix A

The hardware and software interfaces of InertialLAB were designed with a modular and open architecture with the possibility to operate as a stand-alone solution for general human kinematic analysis and to be directly integrated into third-party systems. Appendix A demonstrates the feasibility of the InertialLAB’s integration into a powered exoskeleton, described in [16], for four-fold purposes. First, for real-time gait event detection, as described in [14], to adjust the human-orthosis dynamics for the adaptive impedance control strategy (proposed in [37]). The second purpose includes the real-time estimation of the hip and the knee angles for the powered knee exoskeleton and powered ankle exoskeleton, respectively, for the weight compensation of each exoskeleton through the gravitational compensation strategy. For this purpose, in the knee exoskeleton, we placed two IMUs on the trunk and thigh segments, whereas in the ankle exoskeleton the IMUs were worn on thigh and shank. Third, the InertialLAB can be used with these exoskeletons for the real-time monitoring of the patient’s motor status, and thus, to enabling tailoring the exoskeleton assistance according to this information. Additionally, the system could be used for decision-making (e.g., recognizing desired movements or potential instability).

Figure A1 depicts the hardware interfaces used to achieve a time-effective, deterministic, and ergonomic integration of the InertialLAB into the exoskeletons. We developed an I^2^C interface using an ethernet cable for the communication with the InertialLAB, and a UART interface using the FTDI USB converter for allowing the communication with external processing and storage units.

Moreover, we used the STM32F407VGT as the CPU responsible for (i) data collecting from the exoskeletons and the wearable sensors, (ii) run the software routines implemented in InertialLAB for the joint angle estimation, and (iii) run mechanisms to lighten the weight of the powered devices. The modularity of the software routines implemented in the InertialLAB enabled a prompt integration of these routines into other CPU, limiting the changes to the routine responsible for the peripheric devices’ configuration.

Figure A2 illustrates kinematic signals from the combination of the systems for gait event detection (using the foot angular velocity [14]) and for compensating the ankle exoskeleton’s mass (using the estimated knee angle with IMUs placed on thigh and shank). The absence of a magnetometer beneficiates the InertialLAB’s application in rehabilitation scenarios as exoskeletons and treadmill.

## References

[1] Tao W., Liu T., Zheng R., Feng H. (2012). Gait analysis using wearable sensors. Sensors.

[2] Lambrecht S., Harutyunyan A., Tanghe K., Afschrift M., De Schutter J., Jonkers I. (2017). Real-time gait event detection based on kinematic data coupled to a biomechanical model. Sensors.

[3] González I., Fontecha J., Hervás R., Bravo J. (2015). An Ambulatory System for Gait Monitoring Based on Wireless Sensorized Insoles. Sensors.

[4] Kyrarini M., Wang X., Graser A. Comparison of vision-based and sensor-based systems for joint angle gait analysis. Proceedings of the IEEE International Symposium on Medical Measurements and Applications.

[5] Liu T., Inoue Y., Shibata K. (2009). Development of a wearable sensor system for quantitative gait analysis. Measurement.

[6] Beravs T., Rebersek P., Novak D., Podobnik J., Munih M. Development and validation of a wearable inertial measurement system for use with lower limb exoskeletons. Proceedings of the IEEE-RAS International Conference on Humanoid Robots.

[7] Joukov V., Karg M., Kuli D. Online Tracking of the Lower Body Joint Angles using IMUs for Gait Rehabilitation. Proceedings of the 36th Annual International Conference of the IEEE Engineering in Medicine and Biology Society.

[8] Seel T., Raisch J., Schauer T. (2014). IMU-based joint angle measurement for gait analysis. Sensors.

[9] Ambrožič L., Gorišič M., Šlajpah S., Kamnik R., Munih M. (2014). Wearable sensory system for robotic prosthesis. Int. J. Mech. Control.

[10] Djuric-Jovicic M.D., Jovicic N.S., Popovic D.B. (2011). Kinematics of gait: New method for angle estimation based on accelerometers. Sensors.

[11] Tadano S., Takeda R., Miyagawa H. (2013). Three dimensional gait analysis using wearable acceleration and gyro sensors based on quaternion calculations. Sensors.

[12] Muro-de-la-Herran A., García-Zapirain B., Méndez-Zorrilla A. (2014). Gait analysis methods: An overview of wearable and non-wearable systems, highlighting clinical applications. Sensors.

[13] Kardos S., Balog P., Slosarcik S. (2017). Gait dynamics sensing using IMU sensor array system. Adv. Electr. Electron. Eng..

[14] Figueiredo J., Felix P., Costa L., Moreno J.C., Santos C.P. (2018). Gait Event Detection in Controlled and Real-life Situations: Repeated Measures from Healthy Subjects. IEEE Trans. Neural Syst. Rehabil. Eng..

[15] Figueiredo J., Carvalho S.P., Gonçalves D., Moreno J.C., Santos C.P. (2020). Daily Locomotion Recognition and Prediction: A Kinematic Data-Based Machine Learning Approach. IEEE Access.

[16] Félix P., Figueiredo J., Santos C.P., Moreno J.C. Electronic design and validation of powred knee orthosis system embedded with wearable sensors. Proceedings of the IEEE International Conference on Autonomous Robot Systems and Competitions.

[17] Yang S., Li Q. (2012). Inertial sensor-based methods in walking speed estimation: A systematic review. Sensors.

[18] Xsens Technologies (2013). MVN BIOMECH System: 3D Human Motion Tracking Using Miniature Inertial Sensors.

[19] Brooke J. (1996). SUS: A quick and dirty usability scale. Usability Eval. Ind..

[20] Tucker M.R., Olivier J., Pagel A., Bleuler H., Bouri M., Lambercy O., Millán J.D.R., Riener R., Vallery H., Gassert R. (2015). Control Strategies for Active Lower Extremity Prosthetics and Orthotics: A Review. J. Neuroeng. Rehabil..

[21] InvenSense (2011). MPU-6000 and MPU-6050 Product Specification.

[22] Ribeiro N.F., Rocha L., Santos C.P. (2018). Improvement and Validation of an Inertial System. Under Rev..

[23] Kalman R.E. (1960). A New Approach to Linear Filtering and Prediction Problems 1. J. Fluids Eng..

[24] Amasay T., Zodrow K., Kincl L., Hess J., Karduna A. (2009). Validation of tri-axial accelerometer for the calculation of elevation angles. Int. J. Ind. Ergon..

[25] MATLAB Fit Data with a Shallow Neural Network. https://www.mathworks.com/help/deeplearning/gs/fit-data-with-a-neural-network.html.

[26] MATLAB® Train Regression Models in Regression Learner App. https://www.mathworks.com/help/stats/train-regression-models-in-regression-learner-app.html.

[27] Marquardt D. (1963). An Algorithm for Least-Squares Estimation of Nonlinear Parameters. SIAM J. Appl. Math..

[28] Breiman L., Friedman J.H., Olshen R.A., Stone C.J. (1984). Classification and Regression Trees.

[29] Platt J.C. (1998). Others Sequential minimal optimization: A fast algorithm for training support vector machines. Adv. Kernel Methods Support Vector Learn..

[30] Lebel K., Boissy P., Hamel M., Duval C. (2015). Inertial measures of motion for clinical biomechanics: Comparative assessment of accuracy under controlled conditions-Changes in accuracy over time. PLoS ONE.

[31] Al-Amri M., Nicholas K., Button K., Sparkes V., Sheeran L., Davies J.L. (2018). Inertial measurement units for clinical movement analysis: Reliability and concurrent validity. Sensors.

[32] Zhang J.T., Novak A.C., Brouwer B., Li Q. (2013). Concurrent validation of Xsens MVN measurement of lower limb joint angular kinematics. Physiol. Meas..

[33] Graurock D., Schauer T., Seel T. User-Adaptive Inertial Sensor Network for Feedback-Controlled Gait Support Systems. Proceedings of the Annual International FES Society Conference.

[34] Dumas J.S., Salzman M.C. (2006). Usability Assessment Methods. Rev. Hum. Factors Ergon..

[35] Memedi M., Aghanavesi S., Westin J. A method for measuring Parkinson’s disease related temporal irregularity in spiral drawings. Proceedings of the IEEE-EMBS International Conference on Biomedical and Health Informatics (BHI).

[36] Duong T.T.H., Zhang H., Lynch T.S., Zanotto D. Improving the accuracy of wearable sensors for human locomotion tracking using phase-locked regression models. Proceedings of the International Conference on Rehabilitation Robotics.

[37] Figueiredo J., Félix P., Santos C.P., Moreno J.C. Towards human-knee orthosis interaction based on adaptive impedance control through stiffness adjustment. Proceedings of the International Conference on Rehabilitation Robotics.

